# Supplementary material for: Recombinant expression systems for production of stabilised virus-like particles as next-generation polio vaccines
Source: Nat Commun. 2025 Jan 18;16:831. doi: 10.1038/s41467-025-56118-z (PMC11742952; doi:10.1038/s41467-025-56118-z)
Supplement: Supplementary file 1 — Supplementary Information [file 41467_2025_56118_MOESM1_ESM.pdf]

Recombinant expression systems for production of stabilised virus-like particles as next-generation polio vaccines

Supplementary Tables

	PV1 SC6b (Yeast)		PV1 SC6b (Yeast)	PV1 SC6b (Mammalian)	PV2 SC6b (Mammalian)	PV2 SC6b (Insect)
	DAg	CAg	GPP3 + GSH	CAg	DAg	DAg
	EMD-50064	EMD-50066	EMD-50176	EMD-50112	EMD-50189	EMD-50199
**Data Collection**						
Microscope	Titan Krios G3i (OPIC)		Titan Krios G3i (OPIC)	Titan Krios G3i (OPIC)	Titan Krios IV (eBIC)	Titan Krios IV (eBIC)
Voltage (kV)	300		300	300	300	300
Detector	Gatan K2		Falcon III	Falcon III	Gatan K2	Gatan K3
Recording mode	Counting		Linear	Linear	Counting	Super-resolution
Magnification (×)	47619		129629	129629	47393	47170
Pixel size (Å) (super-resolution)	1.05		1.08	1.08	1.055	1.06 (0.53)
Defocus range (μm)	-2.9 to -0.8		-2.9 to -0.8	-2.9 to -0.8	-2.9 to -0.8	-2.3 to -0.8
Dose rate (e^-/pixel/s)	7.56		53.76	31.03	4.35	14.06
Frames per movie	30		30	25	40	50
Movie exposure time (s)	6.00		0.77	1.27	10.00	2.80
Total electron dose (e^-/Å^2)	41.14		35.49	33.78	39.08	35.04
**Data processing**						
Movies	4282		4467	5104	1331	9379
Initial particles (no.)	4578		159830	7185	18864	11032
Final particles (no.)	1320	2428	23721	6252	18378	3149
Box size (pixels)	400	400	448	400	480	400
Symmetry	I1	I1	I1	I1	I1	I1
Map Resolution (Å)	3.3	3.3	2.8	3.0	2.3	2.6
Map sharpening *B*-factor (Å^2)	-61.8	-76.9	-146.7	-94.0	-67.7	-52.2

Table S1: Cryo-EM data collection and image processing statistics.

	PV1 SC6b (Yeast)		PV1 SC6b (Yeast)	PV1 SC6b (Mammalian)	PV2 SC6b (Mammalian)	PV2 SC6b (Insect)
	DAg	CAg	GPP3 + GSH	CAg	DAg	DAg
	PDB 9EYY	PDB 9EZ0	PDB 9F3Q	PDB 9F0K	PDB 9F59	PDB 9F5P
**Model composition**						
Non-hydrogen atoms	6222	5090	6327	5402	6188	6184
Protein residues	794	654	804	685	772	790
Ligands	PLM: 1		GPP3: 1		SPH: 1	SPH: 1
			GSH: 1			
Waters					123	
**Refinement**						
Resolution (Å)	3.3	3.3	2.8	3.0	2.3	2.6
Map CC[a] (Mask)	0.87	0.85	0.89	0.86	0.89	0.89
Map CC[a] (Volume)	0.86	0.83	0.86	0.84	0.87	0.87
Mean CC[a] (Ligands)	0.78		0.83		0.78	0.80
**RMS deviations**						
Bond lengths (Å)	0.003	0.004	0.003	0.005	0.003	0.005
Bond angles (°)	0.514	0.540	0.544	0.545	0.513	0.573
**Mean B-factor (Å^2)**						
Protein	62.86	69.85	26.95	53.33	24.52	28.48
Ligand	58.97		25.90		24.41	26.66
Water					23.00	
**Validation**						
Molprobity[b] score (percentile)	1.69 (90th)	1.86 (83rd)	0.98 (100th)	1.79 (86th)	1.08 (100th)	1.43 (97th)
Clashscore[b], all atoms (percentile)	6.18 (90th)	6.65 (88th)	1.68 (99th)	3.75 (96th)	2.25 (99th)	4.58 (95th)
Ramachandran favoured (%)	95.03	92.37	97.73	93.53	97.63	96.8
Ramachandran allowed (%)	4.97	7.47	2.27	6.47	2.37	3.2
Ramachandran outliers (%)	0.00	0.16	0.00	0.00	0.00	0.00
Rotamer favoured (outliers) (%)	94.79 (1.01)	95.75 (1.06)	94.70 (0.72)	94.48 (1.84)	97.58 (0.30)	95.85 (0.89)
Cβ deviations >0.25 Å (%)	0.0	0.0	0.0	0.0	0.0	0.0
CaBLAM outliers (%)	1.7	4.2	1.9	2.9	1.1	1.7
CA Geometry outliers (%)	0.52	0.65	0.13	0.93	0.40	0.13
**EMRinger[c] score**	4.54	3.68	5.13	3.80	6.17	4.84

Table S2: Structure refinement and validation for the capsid protein (VP0, VP1, VP3).

[a]Map CC and Mean CC (Ligands) is given for the full icosahedral virus-like particle reconstruction.
[b]Williams *et al.* (2018) Protein Sci 27:293-315.
[c]Barad *et al.* (2015) Nature Methods 12:943–946.

Supplementary figures

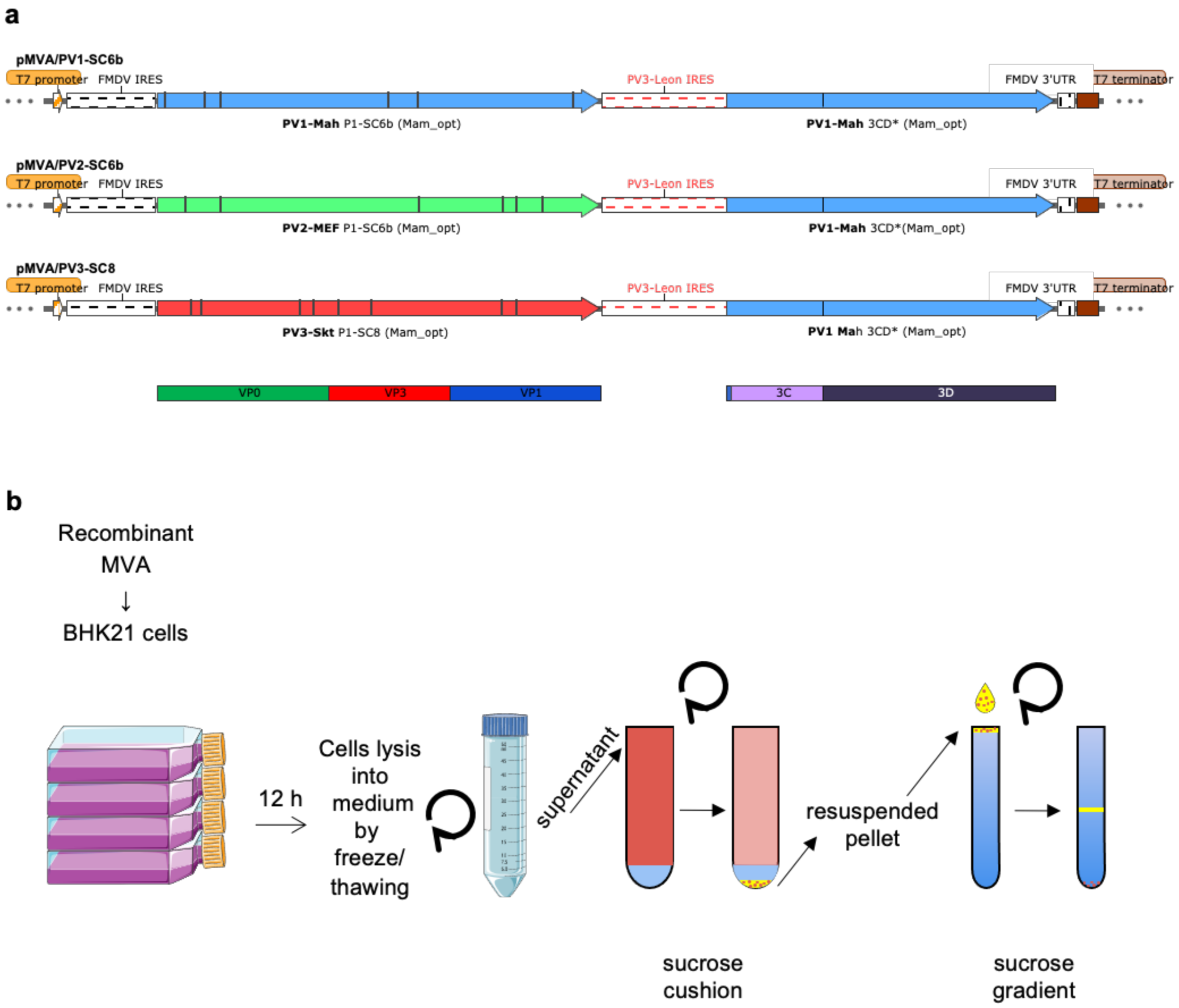

Supplementary Figure 1. Mammalian expression system.

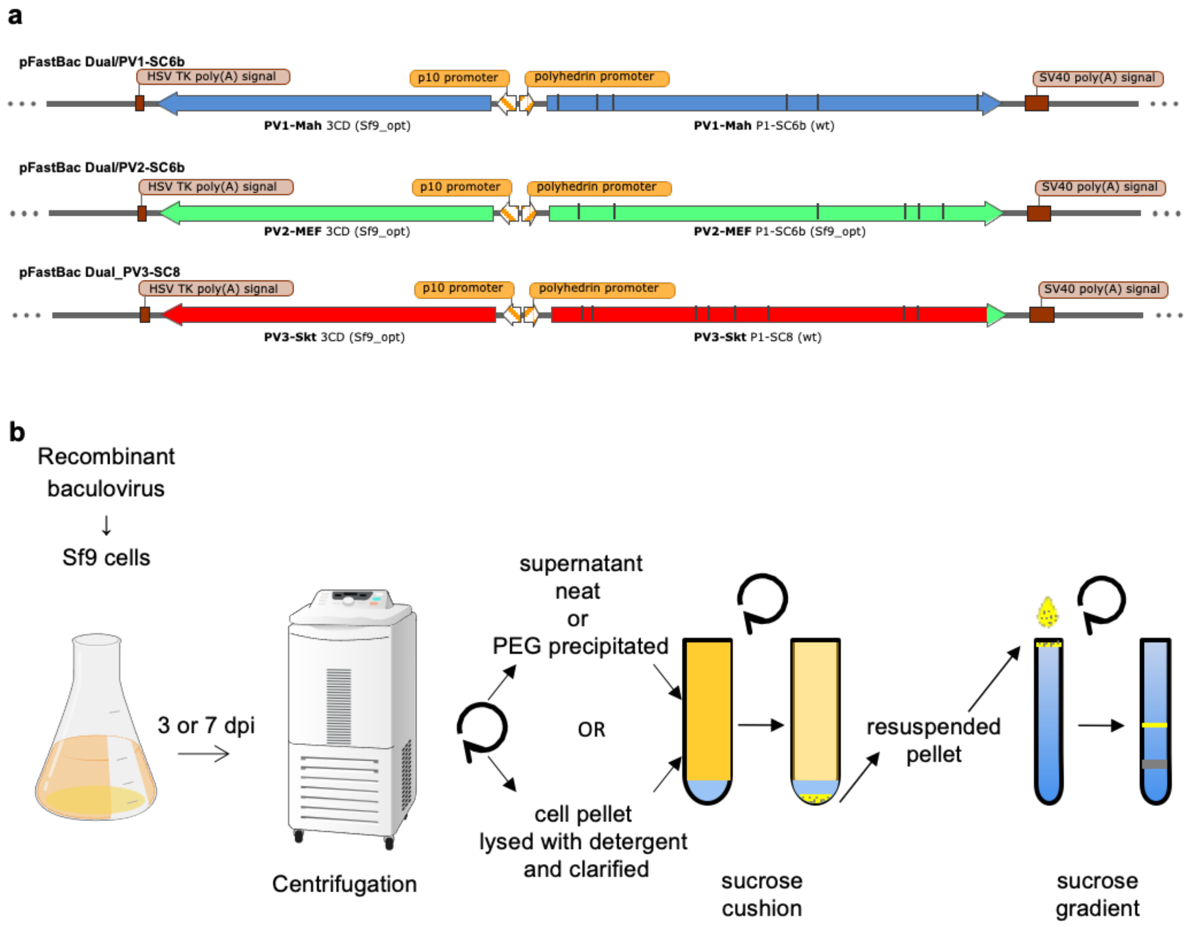

Supplementary Figure 2. Baculovirus expression system.

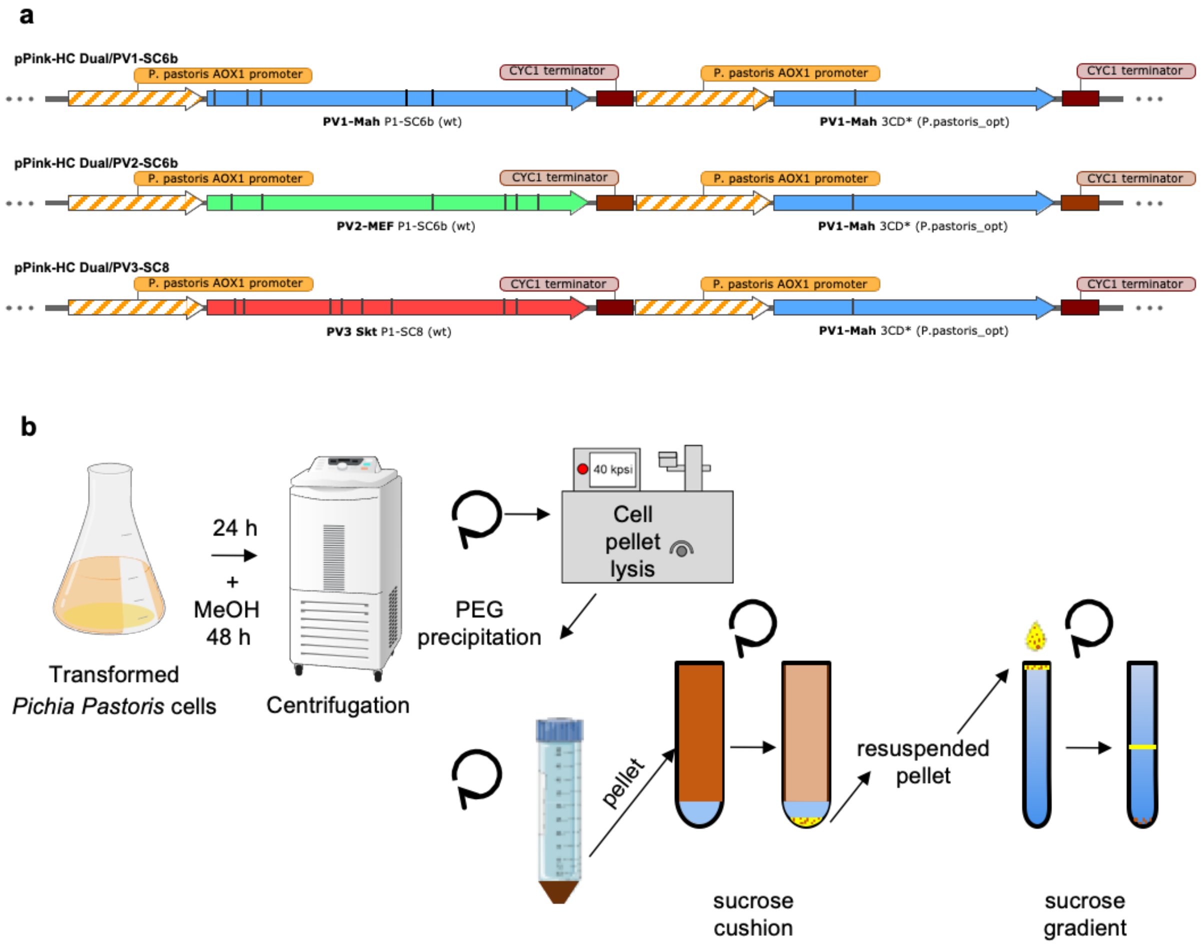

Supplementary Figure 3. Yeast expression system.

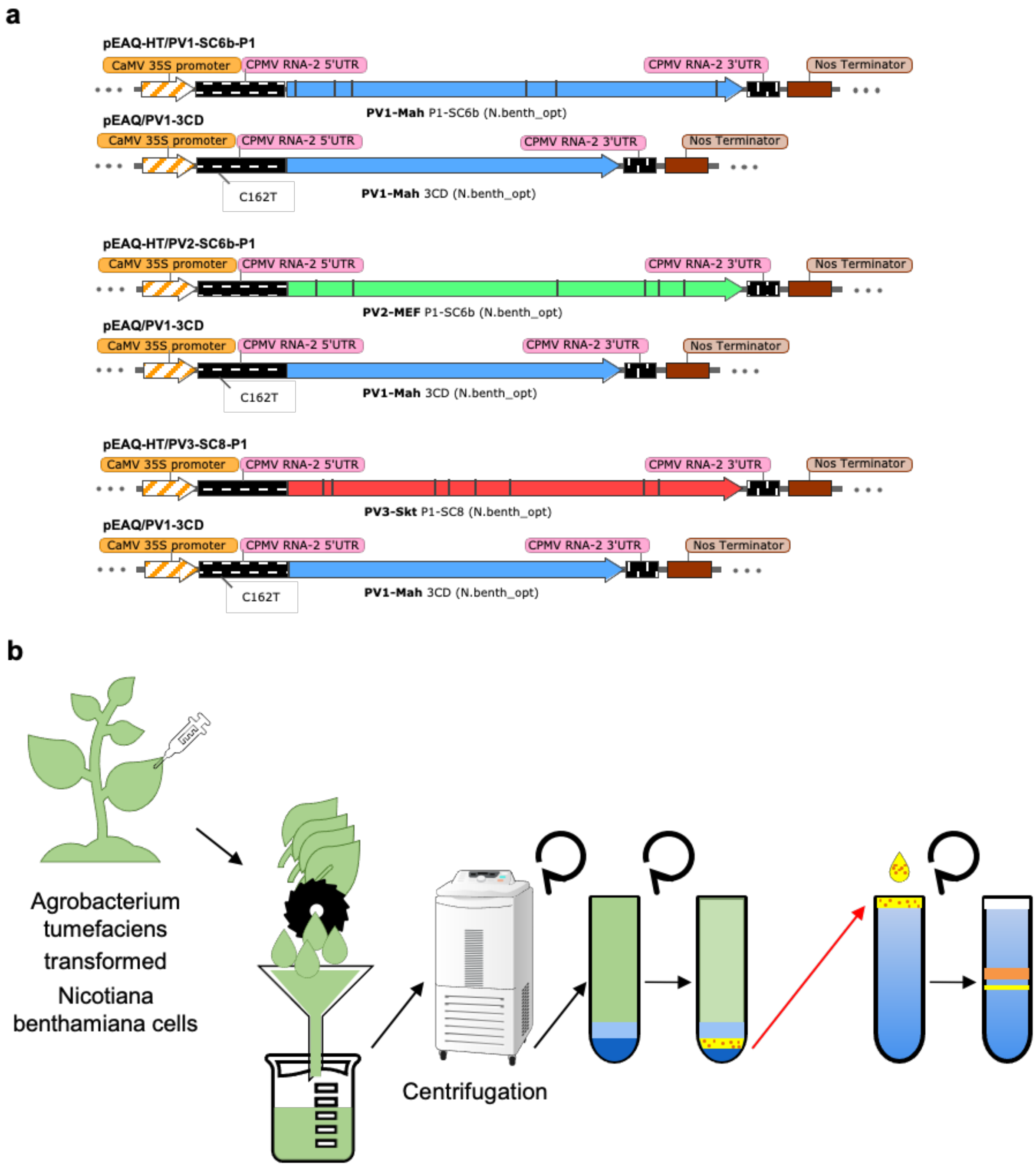

Supplementary Figure 4. Plant expression system.

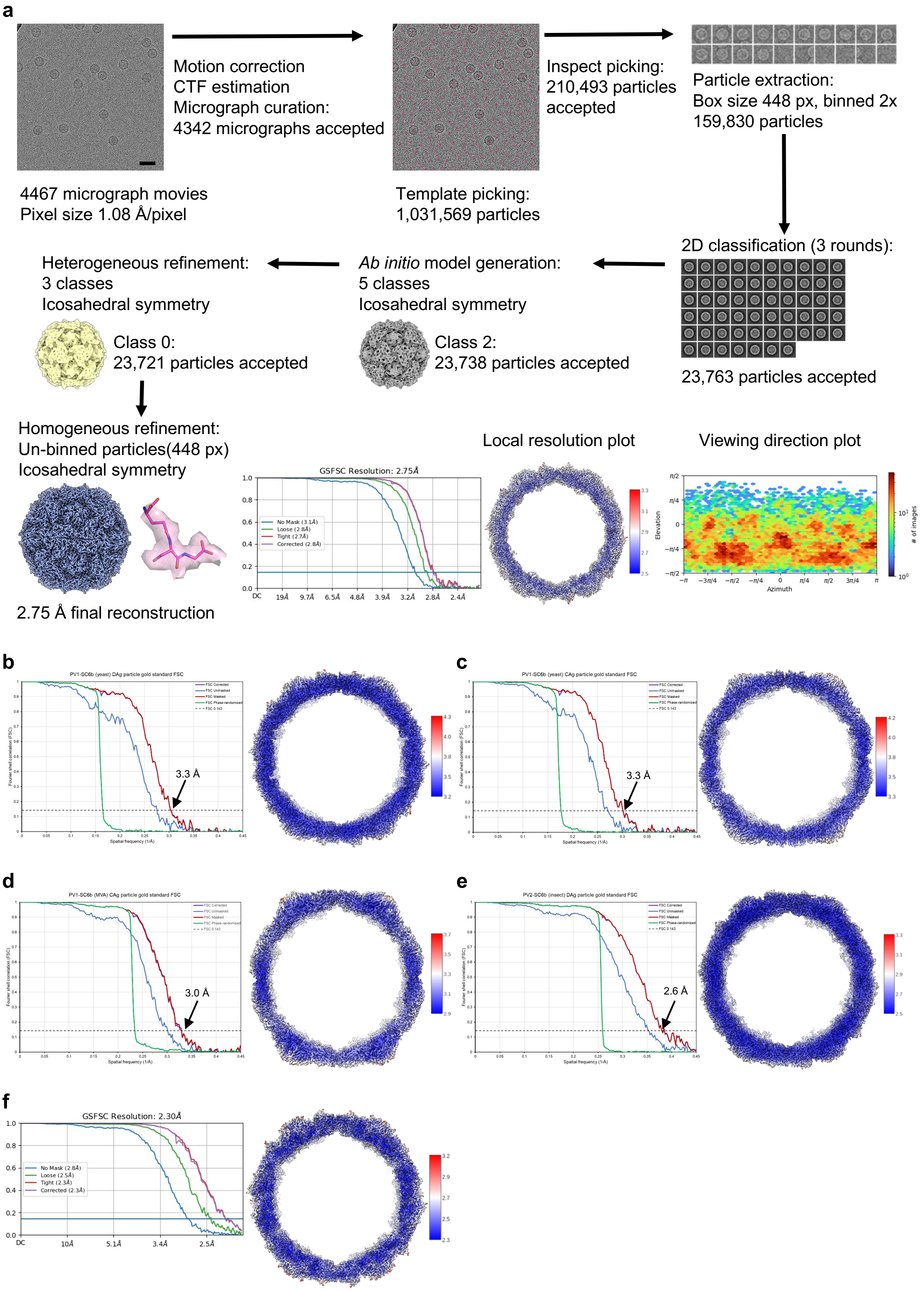

Supplementary Figure 5. Fourier Shell Correlation (FSC) resolution analysis for rsVLP cryoEM reconstructions.

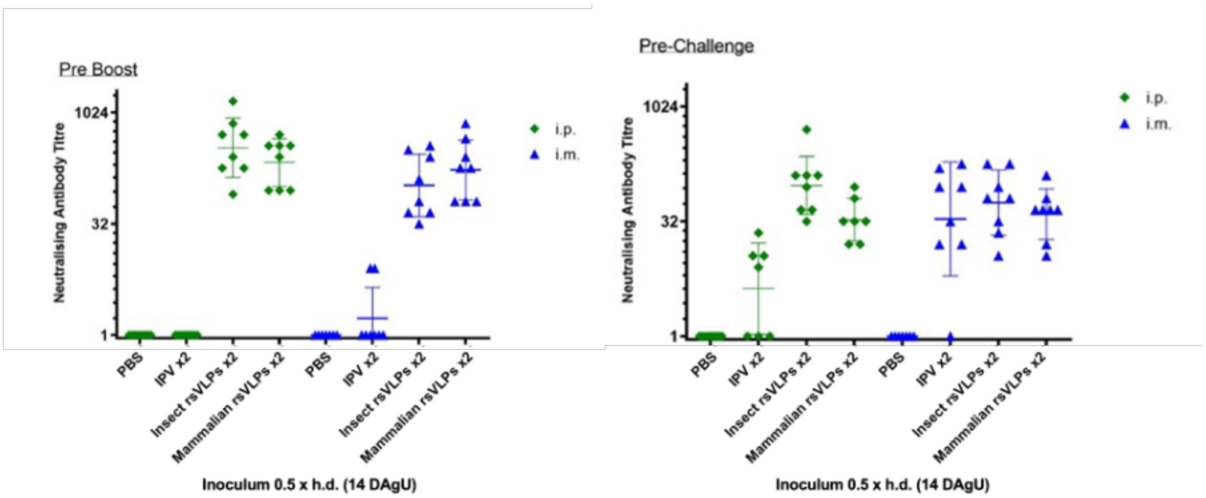

Supplementary Figure 6. Comparison of immunisation routes in TgPVR mice immunised with rsVLPs.

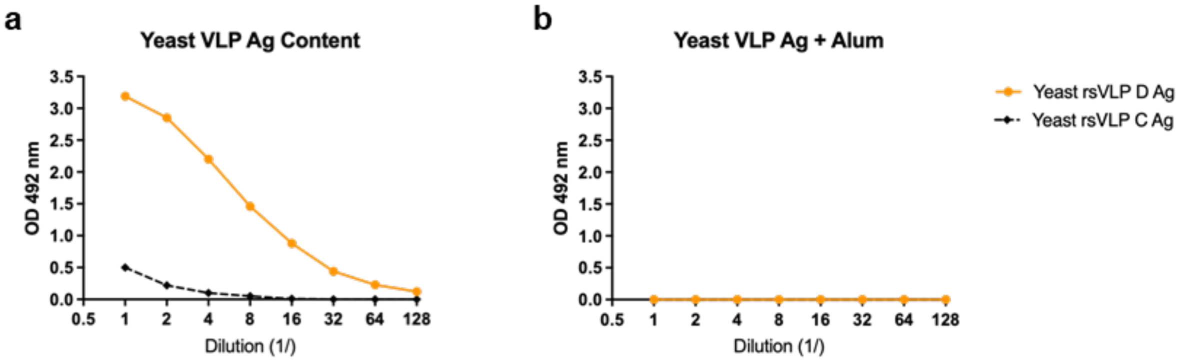

Supplementary Figure 7. rsVLP adjuvant binding.

Supplementary figure legends

Supplementary Figure 1: Mammalian expression system. **a** Each pMVA/PV transfer vector is transfected into primary chicken embryo fibroblasts (CEF) infected with parental MVA for homologous recombination. Mam_opt: codon optimisation for mammalian cells. Bars in the coding sequences of P1 indicate the position of the SC mutations and a bar in 3CD indicates the mutation at the junction between 3C and 3D that results in uncleavable 3CD*. **b** Mammalian cell line BHK-21 is dually infected with each MVA-PV virus alongside MVA-T7 to induce T7 promoter-mediated expression of the rsVLP cassette, followed by downstream purification from the culture supernatant into which the cells content has been released through lysis. The culture-flask-stacked icon and falcon-50mL-empty icon by Servier (smart.servier.com) is licensed under CC-BY 3.0 (bioicons.com).

Supplementary Figure 2: Insect expression system. **a** Each pFastBac Dual/PV transfer vector is recombined into baculovirus shuttle vector, bMON14272, resident in DH10Bac *E. coli* and the resulting recombinant baculoviruses are amplified and expressed in insect cell line Sf9. wt: native viral sequence, Sf9_opt: codon optimisation for Sf9 insect cells. Bars in the coding sequences of P1 indicate the position of the SC mutations in P1. **b** Between 3 and 7 dpi, cultures are centrifuged and rsVLPs purified separately from the resulting cell pellet and supernatant. The centrifuge-big icon by DBCLS (togotv.dbcls.jp/en/pics.html) is licensed under CC-BY 4.0 (bioicons.com). The erlenmeyer_filled icon by KeHan (github.com/kehantan) is licensed under CC0 (bioicons.com).

Supplementary Figure 3: Yeast expression system. **a** Each pPink-HC-Dual/PV vector is transfected into yeast strain PichiaPink™ (selection through *ADE2* gene complementation) and expression of the PV genes controlled by *AOX1* promoters is induced by methanol. wt: native viral sequence, P. pastoris_opt: codon optimisation for *Pichia pastoris*. Bars in the coding sequences of P1 indicate the position of the SC mutations and a bar in 3CD indicates the mutation at the junction between 3C and 3D that results in uncleavable 3CD*. **b** Highly expressing PV VLP *Pichia pastoris* clones are grown to high density for 24 h in YPD medium prior to induction through the addition of methanol containing medium, YPM for 48 h when cell pellets are collected by centrifugation, followed by downstream purification. The centrifuge-big icon by DBCLS (togotv.dbcls.jp/en/pics.html) is licensed under CC-BY 4.0 (bioicons.com). The erlenmeyer_filled icon by KeHan (github.com/kehantan) is licensed under CC0 (bioicons.com). The falcon-50mL-empty icon by Servier (smart.servier.com) is licensed under CC-BY 3.0 (bioicons.com).

Supplementary Figure 4: Plant expression system. **a** pEAQ-HT/PVx-P1 and pEAQ/PV1-3CD plasmids are independently transformed into *Agrobacterium tumefaciens* strain LBA4404 and after amplification each pair of recombinant bacteria is mixed prior to infiltration into the leaves of *Nicotiana benthamiana* plants. N.benth_opt: codon optimisation for *Nicotiana benthamiana* plant cells. Bars in the coding sequences of P1 indicate the position of the SC mutations. **b** The leaves are harvested 6 dpi and homogenised in a blender for rsVLP purification from the resulting sap. The centrifuge-big icon by DBCLS (togotv.dbcls.jp/en/pics.html) is licensed under CC-BY 4.0 (bioicons.com).

Supplementary Figure 5: Data processing workflow and local resolution analysis of PV rsVLP cryoEM reconstructions. **a** Representative data processing scheme for single particle analysis of the PV1-SC6b$^{GPP3+GSH}$ rsVLP performed in CryoSPARC v4.2.1. A micrograph of the sample is shown top left, scale bar: 50 nm. A final consensus 3D reconstruction was obtained by homogeneous refinement of 23,721 particles, to a resolution of 2.8 Å. Glutathione ligand is shown as a magenta stick model fitted to the cryoEM map (threshold 1.0 σ). Gold standard Fourier shell correlation (GSFSC) curves and viewing direction distribution plots are shown. Local resolution analysis of the final electron potential map is shown as a central slice through the rsVLP viewed along the icosahedral two-fold axis, and the distribution of local resolution (in Å) is coloured from blue to red according to the colour key. **b-e** left panels, Fourier shell correlation (FSC) calculated between two independent half sets of data as a function of spatial frequency is plotted for the (**b**) PV1-SC6b D Ag particle (yeast), (**c**) PV1-SC6b C Ag particle (yeast), (**d**) PV1-SC6b C Ag particle (MVA) and (**e**) PV2-SC6b D Ag particle (baculovirus) reconstructions performed in RELION. FSC is plotted for the original unmasked half-maps (blue) and masked half-maps that had density corresponding to solvent removed (red). FSC is also shown for phase-randomized half-maps (green) used to compensate for possible effects of the masking procedure before calculating the final corrected FSC (purple). Good agreement between the masked and corrected curves indicated no adverse effects from the masking. The resolution at which the corrected curve drops below the FSC=0.143 threshold (black dashed line) is indicated with an arrow. The right panels show local resolution analysis of the final cryoEM electron potential maps for each rsVLP as assessed by RELION local resolution estimation. A central slice through each rsVLP is viewed and coloured as in (**a**). **f** Left panel, GSFSC curve for the PV2-SC6b (MVA) reconstruction, from CryoSPARC. Right panel, CryoSPARC assessed local resolution analysis depicted as in (**a**).

Supplementary Figure 6: Comparison of immunisation routes in TgPVR mice immunised with rsVLPs. a Groups of 8 mice received 2 doses of rsVLPs produced in mammalian or insect cells (day 0 and 14) through intraperitoneal (i.p - green) or intramuscular immunisation routes (i.m - blue). Neutralisation titres prior to boost and on the day of challenge were determined.

Supplementary Figure 7: rsVLP adjuvant binding. a rsVLP samples were tested for D and C Ag content (orange and black, respectively) using serotype-specific monoclonal antibodies before the addition of 1/10th volume of Alhydrogel (2%, InvivoGen). **b** Following adsorption and 30 min agitation, the resulting supernatant was tested for D and C Ag content.
