# Supplementary material for: Recombinant expression systems for production of stabilised virus-like particles as next-generation polio vaccines
Source: Nat Commun. 2025 Jan 18;16:831. doi: 10.1038/s41467-025-56118-z (PMC11742952; doi:10.1038/s41467-025-56118-z)
Supplement: Supplementary file 3 — Reporting Summary [file 41467_2025_56118_MOESM3_ESM.pdf]

nature portfolio

Corresponding author(s): David J Rowlands, Nicola J Stonehouse, George P Lomonossoff, Andrew J Macadam, David I Stuart, Elizabeth E Fry

Last updated by author(s): Dec 21, 2024

Reporting Summary

Nature Portfolio wishes to improve the reproducibility of the work that we publish. This form provides structure for consistency and transparency in reporting. For further information on Nature Portfolio policies, see our Editorial Policies and the Editorial Policy Checklist.

Statistics

For all statistical analyses, confirm that the following items are present in the figure legend, table legend, main text, or Methods section.

n/a	Confirmed	
☐	☒	The exact sample size (*n*) for each experimental group/condition, given as a discrete number and unit of measurement
☐	☒	A statement on whether measurements were taken from distinct samples or whether the same sample was measured repeatedly
☒	☐	The statistical test(s) used AND whether they are one- or two-sided<br>*Only common tests should be described solely by name; describe more complex techniques in the Methods section.*
☒	☐	A description of all covariates tested
☒	☐	A description of any assumptions or corrections, such as tests of normality and adjustment for multiple comparisons
☐	☒	A full description of the statistical parameters including central tendency (e.g. means) or other basic estimates (e.g. regression coefficient) AND variation (e.g. standard deviation) or associated estimates of uncertainty (e.g. confidence intervals)
☒	☐	For null hypothesis testing, the test statistic (e.g. *F*, *t*, *r*) with confidence intervals, effect sizes, degrees of freedom and *P* value noted<br>*Give P values as exact values whenever suitable.*
☒	☐	For Bayesian analysis, information on the choice of priors and Markov chain Monte Carlo settings
☒	☐	For hierarchical and complex designs, identification of the appropriate level for tests and full reporting of outcomes
☒	☐	Estimates of effect sizes (e.g. Cohen's *d*, Pearson's *r*), indicating how they were calculated

Our web collection on statistics for biologists contains articles on many of the points above.

Software and code

Policy information about availability of computer code

Data collection	Thermo Fisher Scientific EPU software for microscope control and data acquisition.
Data analysis	RELION version 3.1, CryoSPARC v4.2.1 and crYOLO version 1.5 for cryoEM data processing, Coot version 0.9.6 for model building, Namdinator molecular dynamics flexible fitting software (https://namdinator.au.dk/), Phenix software version 1.20.1-4487 for model refinement and data analysis, UCSF Chimera version 1.16 and UCSF ChimeraX version 1.3 for structure analysis and graphical rendering, PyMOL version 2.5.2 for graphical rendering. Immunogenicity data was analysed using Prism version 10.4.1.

For manuscripts utilizing custom algorithms or software that are central to the research but not yet described in published literature, software must be made available to editors and reviewers. We strongly encourage code deposition in a community repository (e.g. GitHub). See the Nature Portfolio guidelines for submitting code & software for further information.

Data

Policy information about availability of data

All manuscripts must include a data availability statement. This statement should provide the following information, where applicable:

- Accession codes, unique identifiers, or web links for publicly available datasets
- A description of any restrictions on data availability
- For clinical datasets or third party data, please ensure that the statement adheres to our policy

The atomic coordinates for the cryoEM structures in this study have been submitted to the Protein Data Bank under the following accession codes (PDB ID): PV1-SC6b yeast D Ag particle (9EYY), PV1-SC6b yeast C Ag particle (9EZ0), PV1-SC6bGPP3+GSH yeast (9F3Q), PV1-SC6b mammalian C Ag particle (9F0K), PV2-SC6b

mammalian (9F59), PV2-SC6b insect (9F5P). The cryoEM electron potential maps have been deposited in the Electron Microscopy Data Bank under the following accession codes (EMD ID): PV1-SC6b yeast D Ag particle (EMD-50064), PV1-SC6b yeast C Ag particle (EMD-50066), PV1-SC6bGPP3+GSH yeast (EMD-50176), PV1-SC6b mammalian C Ag particle (EMD-50112), PV2-SC6b mammalian (EMD-50189), PV2-SC6b insect (EMD-50199). The source data underlying Fig. 4-6 and supplementary figures 6 and 7 are provided with this paper. The data generated and/or analysed during the current study are available from the corresponding authors on reasonable request.

Research involving human participants, their data, or biological material

Policy information about studies with human participants or human data. See also policy information about sex, gender (identity/presentation), and sexual orientation and race, ethnicity and racism.

Reporting on sex and gender	*Use the terms sex (biological attribute) and gender (shaped by social and cultural circumstances) carefully in order to avoid confusing both terms. Indicate if findings apply to only one sex or gender; describe whether sex and gender were considered in study design; whether sex and/or gender was determined based on self-reporting or assigned and methods used.*<br>*Provide in the source data disaggregated sex and gender data, where this information has been collected, and if consent has been obtained for sharing of individual-level data; provide overall numbers in this Reporting Summary. Please state if this information has not been collected.*<br>*Report sex- and gender-based analyses where performed, justify reasons for lack of sex- and gender-based analysis.*
Reporting on race, ethnicity, or other socially relevant groupings	*Please specify the socially constructed or socially relevant categorization variable(s) used in your manuscript and explain why they were used. Please note that such variables should not be used as proxies for other socially constructed/relevant variables (for example, race or ethnicity should not be used as a proxy for socioeconomic status).*<br>*Provide clear definitions of the relevant terms used, how they were provided (by the participants/respondents, the researchers, or third parties), and the method(s) used to classify people into the different categories (e.g. self-report, census or administrative data, social media data, etc.)*<br>*Please provide details about how you controlled for confounding variables in your analyses.*
Population characteristics	*Describe the covariate-relevant population characteristics of the human research participants (e.g. age, genotypic information, past and current diagnosis and treatment categories). If you filled out the behavioural & social sciences study design questions and have nothing to add here, write "See above."*
Recruitment	*Describe how participants were recruited. Outline any potential self-selection bias or other biases that may be present and how these are likely to impact results.*
Ethics oversight	*Identify the organization(s) that approved the study protocol.*

Note that full information on the approval of the study protocol must also be provided in the manuscript.

Field-specific reporting

Please select the one below that is the best fit for your research. If you are not sure, read the appropriate sections before making your selection.

☒ Life sciences ☐ Behavioural & social sciences ☐ Ecological, evolutionary & environmental sciences

For a reference copy of the document with all sections, see nature.com/documents/nr-reporting-summary-flat.pdf

Life sciences study design

All studies must disclose on these points even when the disclosure is negative.

Sample size	The sample sizes for the transgenic mice and rat tests were determined by previous work which establishing poliovirus vaccine batch release assays. The WHO standard operating procedure, including sample size and statistical analysis for transgenic mice susceptible to poliovirus can be found here: https://cdn.who.int/media/docs/default-source/biologicals/vaccine-standardization/poliomyelitis/polio_sop_tgmnvt_sopv7_30_june2015_clean2.pdf. The paper describing the establishment of the rat bioassay for IPV can be found here: https://www.edqm.eu/documents/52006/123862/bsp021-ipv-bioassay1.pdf/2219b777-1928-be62-6f84-231b1b7189b3 which builds on the work of van Steenis et al, as specified in the manuscript (Ref. 58)
Data exclusions	No data has been excluded from this study
Replication	The antigenicity of rsVLPs was ratified between different institutes throughout the study, ensuring the correct immunisation dose was used in our immunogenicity trials. These experiments have been successfully replicated across the different expression systems.
Randomization	Animals were randomly assigned to different experimental groups through a random number generator
Blinding	The inocula used for each animal was anonymised, therefore the person administering the dose was blinded to the group allocation of each animal in the experiment.

Reporting for specific materials, systems and methods

We require information from authors about some types of materials, experimental systems and methods used in many studies. Here, indicate whether each material, system or method listed is relevant to your study. If you are not sure if a list item applies to your research, read the appropriate section before selecting a response.

Materials & experimental systems

n/a	Involved in the study
	☒ Antibodies
	☒ Eukaryotic cell lines
☒	Palaeontology and archaeology
	☒ Animals and other organisms
☒	Clinical data
☒	Dual use research of concern
	☒ Plants

Methods

n/a	Involved in the study
☒	ChIP-seq
☒	Flow cytometry
☒	MRI-based neuroimaging

Antibodies

Antibodies used	D Ag specific monoclonal antibody Type 1, MAb 234, supplied by NIBSC. Catalogue Number 234, Dilution 1:100<br>D Ag specific monoclonal antibody Type 2, MAb 1050, supplied by NIBSC. Catalogue Number 1050, Dilution 1:100<br>D Ag specific monoclonal antibody Type 3, MAb 520, supplied by NIBSC. Catalogue Number 520, Dilution 1:100<br>C Ag specific monoclonal antibody Type 1, MAb 1588, supplied by NIBSC (not for sale), Dilution 1:100<br>C Ag specific monoclonal antibody Type 3, MAb 517, supplied by NIBSC (not for sale), Dilution 1:100<br>Anti-Poliovirus Blend Antibody, clones 583-G8-G2-A4, 591-B1-H7-D1, and 613-F1-B5-E5 purchased from Sigma-Aldrich. Catalogue Number MAB8566 and used at 1:1000 for immunoblot detection.
Validation	Antibodies used in D Ag specific ELISA have been validated by WHO Collaborative studies for use in IPV lot release assays.<br>Antibodies used in C Ag specific ELISA have been validated in-house by NIBSC/MHRA using the same protocols.<br>Anti-Poliovirus Blend Antibody, clones 583-G8-G2-A4, 591-B1-H7-D1, and 613-F1-B5-E5 purchased from Sigma-Aldrich. Catalogue Number MAB8566 has been validated for immunofluorescence on the manufacturer's website (https://www.sigmaaldrich.com/GB/en/product/mm/mab8566) and previously validated in immunoblot assays in reference 42 of the manuscript.

Eukaryotic cell lines

Policy information about cell lines and Sex and Gender in Research

Cell line source(s)	BHK-21 cells were sourced from the Pirbright Institute. GMK cells were sourced from the American Type Culture Collection (ATCC).<br>Insect cell line Sf9 was purchased from ThermoFisher Scientific. Catalogue number: 11496015<br>Yeast cell line Pichia pastoris (Pichia Pink Strain 1: ADE2) was purchased from ThermoFisher Scientific. Catalogue number: A11154
Authentication	None of the cell lines were authenticated
Mycoplasma contamination	All cell lines tested negative for Mycoplasma
Commonly misidentified lines (See ICLAC register)	No commonly misidentified cell lines were used in this study

Animals and other research organisms

Policy information about studies involving animals; ARRIVE guidelines recommended for reporting animal research, and Sex and Gender in Research

Laboratory animals	TgPVR mice aged between 6-8 weeks were supplied from a colony maintained at MHRA.<br>Wistar rats aged between 6-12 weeks were supplied by Charles River UK Ltd.<br>We abide by the Animals (Scientific Procedures) Act's code of Practice Code of Practice: Animals. These guidelines stipulate rules on lighting, temp, humidity, air changes and cage sizes. Lighting = 12 hours light/12 hours dark, changes at 6:30 am and 6:30 pm with 30 mins of half light. Temperature = maintained between 20-24C Humidity = 45-65% Floor area = 530cm2
Wild animals	*Provide details on animals observed in or captured in the field; report species and age where possible. Describe how animals were caught and transported and what happened to captive animals after the study (if killed, explain why and describe method; if released, say where and when) OR state that the study did not involve wild animals.*
Reporting on sex	Immunisation challenge experiments in TgPVR mice used equal numbers of male and female mice.<br>Immunogenicity assays in Wistar rats used only female animals, as specified in ref 59.
Field-collected samples	*For laboratory work with field-collected samples, describe all relevant parameters such as housing, maintenance, temperature, photoperiod and end-of-experiment protocol OR state that the study did not involve samples collected from the field.*

Ethics oversight	All animal experiments were performed under licenses granted by the UK Home Office under the Animal (Scientific Procedures) Act 1986 revised 2013 and reviewed by the internal NIBSC Animal Welfare and Ethics Review Board.

Note that full information on the approval of the study protocol must also be provided in the manuscript.

Dual use research of concern

Policy information about dual use research of concern

Hazards

Could the accidental, deliberate or reckless misuse of agents or technologies generated in the work, or the application of information presented in the manuscript, pose a threat to:

No	Yes	
☒	☐	Public health
☒	☐	National security
☒	☐	Crops and/or livestock
☒	☐	Ecosystems
☒	☐	Any other significant area

Experiments of concern

Does the work involve any of these experiments of concern:

No	Yes	
☒	☐	Demonstrate how to render a vaccine ineffective
☒	☐	Confer resistance to therapeutically useful antibiotics or antiviral agents
☒	☐	Enhance the virulence of a pathogen or render a nonpathogen virulent
☒	☐	Increase transmissibility of a pathogen
☒	☐	Alter the host range of a pathogen
☒	☐	Enable evasion of diagnostic/detection modalities
☒	☐	Enable the weaponization of a biological agent or toxin
☒	☐	Any other potentially harmful combination of experiments and agents

Plants

Seed stocks	We used wild-type Nicotiana benthamiana and the seeds are propagated and stored at the John Innes Centre
Novel plant genotypes	No novel genotypes were produced for this work.
Authentication	*Describe any authentication procedures for each seed stock used or novel genotype generated. Describe any experiments used to assess the effect of a mutation and, where applicable, how potential secondary effects (e.g. second site T-DNA insertions, mosiacism, off-target gene editing) were examined.*
